# The Dermatophyte *Trichophyton rubrum* Induces Neutrophil Extracellular Traps Release by Human Neutrophils

**DOI:** 10.3390/jof8020147

**Published:** 2022-01-31

**Authors:** Ana Paula Carvalho Reis, Giovanna Azevedo Celestrino, Mariana Villas Bôas Igoa, Thais Martins Jesus, Tábata Takahashi França, Daniel Valério Silva Moreira, Paula Ordonhez Rigato, Paula Keiko Sato, Antonio Condino-Neto, Irene L. Noronha, Luciane Alarcão Dias-Melicio, Pritesh Jaychand Lalwani, Gil Benard, Maria Gloria Teixeira Sousa

**Affiliations:** 1 Laboratory of Medical Mycology LIM-53, Clinical Dermatology Division, Hospital das Clínicas FMUSP, Faculdade de Medicina FMUSP, Institute of Tropical Medicine, University of São Paulo, São Paulo 05403-000, Brazil; aninhareis97@gmail.com (A.P.C.R.); acgiovanna01@gmail.com (G.A.C.); marianaigoa1@hotmail.com (M.V.B.I.); thapmartins@gmail.com (T.M.J.); danielvalerio.sm@gmail.com (D.V.S.M.); bengil60@gmail.com (G.B.); 2 Laboratory of Cellular, Genetic and Molecular Nephrology, Division of Nephrology, University of São Paulo School of Medicine, São Paulo 05403-000, Brazil; irenenor@usp.br; 3 Department of Immunology, Institute of Biomedical Sciences, University of São Paulo, São Paulo 05403-000, Brazil; tabata.t.franca@gmail.com (T.T.F.); antoniocondino@gmail.com (A.C.-N.); 4 Centro de Imunologia, Instituto Adolfo Lutz, São Paulo 01246-000, Brazil; paula.rigato@ial.sp.gov.br; 5 Laboratory of Medical Investigation in Immunology (LIM-48), Hospital das Clinicas HCFMUSP, Faculdade de Medicina, Universidade de Sao Paulo, São Paulo 05403-000, Brazil; paulaksato@yahoo.com; 6 Department of Pathology, Botucatu Medical School, UNESP-São Paulo State University, Botucatu 18618-687, Brazil; dias.melicio@unesp.br; 7 Leonidas e Maria Deane Institute, Fiocruz Amazônia, Manaus 69057-070, Brazil; lalwanipritesh@yahoo.com

**Keywords:** dermatophytosis, *Trichophyton rubrum*, neutrophils, neutrophil extracellular traps, fungal killing, innate immunity

## Abstract

Neutrophils are the first leukocytes recruited to the site of infection and are thought to be responsible for fungal elimination from the skin such as dermatophytes. Neutrophils are able to secrete reactive oxygen species (ROS) and neutrophil extracellular traps (NETs) that can kill different fungi, including *Aspergillus*, spp., *Candida albicans*, and *Phialophora verrucosa*. However, NET production in response to *Trichophyton rubrum*, the main etiologic agent of dermatophytosis, has yet to be studied. We demonstrated that human neutrophils produce NETs against different morphotypes of *T. rubrum* in a dose-dependent manner and NET formation is dependent on ROS production. In addition, ROS production by human neutrophils in response to *T. rubrum* is dependent on NADPH oxidase, but not on fungal viability. NETs mediated killing of *T. rubrum.* Collectively, these results demonstrate that *T. rubrum* was able to trigger the production of NETs, suggesting that these extracellular structures may represent an important innate immune effector mechanism controlling physiological response to *T. rubrum* infection.

## 1. Introduction

Dermatophytoses are superficial fungal infections of keratinized structures, and the main etiological agent in humans is *Trichophyton rubrum*. It is considered an anthropophilic dermatophyte, thus well adapted to humans, usually causing mild inflammation and cutaneous lesions [1]. Yet, although causing primarily superficial infections, in immunocompromised patients, it can disseminate beyond the superficial layers, potentially leading to a systemic infection with deep organ involvement, namely deep dermatophytosis [2].

Deep dermatophytosis is rare and is often characterized by inflammatory cell infiltration, mostly composed of neutrophils and macrophages [3,4]. Neutrophils are the most abundant leukocytes in the blood, being the first cells to arrive at an infection site [5]. Their main antifungal function is due to their phagocytosis ability and their potent antimicrobial arsenal composed of antimicrobial peptides, neutrophil-specific proteolytic enzymes, as well as their production of reactive oxygen species (ROS) [6,7].

In addition, these cells can also produce neutrophil extracellular traps (NETs), characterized by the release of their chromatin contents to the extracellular environment, creating a scaffold for association with different cytoplasmic proteins, such as histones and enzymes, which creates a mesh-like structure that helps in the entrapment and elimination of pathogens. Although different stimuli can lead to NET initiation, the production of ROS by the enzymatic complex NADPH oxidase is an important pathway [8,9,10].

NET release can be triggered by several stimuli, including a diverse set of pathogenic fungi as *Cryptococcus neoformans*, *Candida albicans*, *Aspergillus fumigatus*, *Paracoccidioides brasiliensis*, and *Phialophora verrucose* [11,12]. However, no data are currently available on the role of NETs against *T. rubrum*. This study aimed to elucidate the ability of different morphotypes of *T. rubrum* to induce ROS production and NET release by human neutrophils.

## 2. Results

### 2.1. T. rubrum Induces Neutrophil Extracellular Traps (NETs) Release in a Dose-Dependent Manner

**Figure 1:**
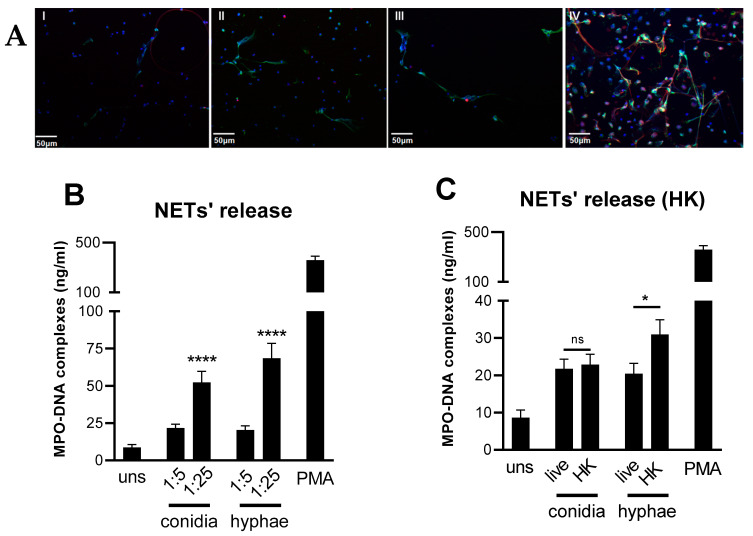
*T. rubrum* promotes neutrophil extracellular traps (NETs) formation by neutrophils. Human neutrophils were isolated and infected with *T. rubrum* conidia for 3 h at 37 °C. Phorbol myristate acetate (PMA) of 100 nM was used as a positive control for NET formation. (**A**) (I–IV) Immunostaining of NET components (green, myeloperoxidase; red, histone; DNA Hoechst, blue). Scale bar = 50 µm. (I) Unstimulated neutrophils incubated for 180 min do not show NETs. Neutrophils stimulated with *T. rubrum* conidia (II), hyphae (III), or PMA (IV) for 180 min showed NETs. Images were captured using a Nikon Eclipse 80i. Scale bar: 50 µm. (**B**) NET formation is dose dependent. Neutrophils were incubated for 3 h at 37 °C with different ratios of *T. rubrum* morphotypes (conidia or hyphae) (multiplicity of infection (MOI) 1:5 or 1:25), and myeloperoxidase (MPO)-DNA complexes were measured in the culture supernatants. (**C**) Fungal viability is not essential for NET induction. Neutrophils were incubated for 3 h at 37 °C with live or heat-killed (HK). *T. rubrum* morphotypes and MPO-DNA complexes were measured in the culture supernatants. NET quantification was performed using the MPO-DNA PicoGreen assay. Data are presented as the mean±SEM of three independent experiments. One-way ANOVA and Bonferroni’s post-test: ns, not significant; * *p* < 0.05; **** *p* < 0.0001.

NETs cause tissue damage by releasing DNA and granular proteins, such as myeloperoxidase (MPO), elastase, and histone, triggering apoptosis and fibrosis. Since they can be visualized as extracellular fibers of DNA colocalizing with MPO and citrullinated histone H3 [8,13], we investigated whether *T. rubrum* conidia or hyphae promote NETs’ release in human neutrophils (Figure 1). We also used phorbol myristate acetate (PMA) as a positive control for NET induction. Indeed, we could observe structures compatible with NETs in neutrophils incubated with both morphotypes of *T. rubrum* (Figure 1A). We further confirmed these results by measuring MPO-DNA complexes in the supernatants (Figure 1B), detecting an increased concentration of complexes in infected cells. We also noticed that the formation of NETs occurs in a dose-dependent manner.

Next, we questioned if fungal viability was a prerequisite for NETs’ induction. Thus, we challenged the neutrophils with live or heat-killed (HK) structures and investigated their response (Figure 1C). While fungal inactivation did not reduce NET formation, we observed an increased response to HK hyphae, which could be due to changes in the exposure of antigens (mannoproteins or altered β-glucans) after the heating treatment, similar to previous reports for *C. albicans*.

### 2.2. Reactive Oxygen Species (ROS) Production by Neutrophils against T. rubrum

**Figure 2:**
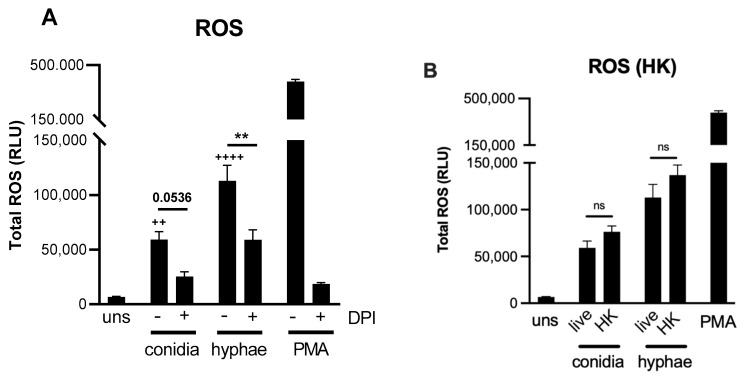
*T. rubrum* morphotypes induce ROS production by human neutrophils. Neutrophils were incubated with *T. rubrum* conidia or hyphae at MOI (1:5) for 2 h at 37 °C. Neutrophils were pre-treated with DPI for 30 min when required. (**A**) Respiratory bursts from neutrophils treated with various stimuli were analyzed by using luminol-enhanced chemiluminescence; values are expressed as relative light units (RLU). (**B**) ROS production with live or heat-killed fungi. Data shown as the mean ± SEM. One-way ANOVA and Bonferroni’s post-test: ns, not significant; ^**++**^ *p* < 0.01; **^++++^** *p* < 0.0001 (vs. unstimulated group); ** *p* < 0.01.

ROS production by neutrophils is a well-described effector mechanism that confers resistance against opportunistic pathogens such as *Candida albicans* and *Paracoccidioides brasiliensis* [14,15]. We previously showed that chronic widespread dermatophytosis patients’ neutrophils presented reduced hydrogen peroxide (H_2_O_2_) production when compared with healthy donors [16]. Next, we assessed whether *T. rubrum* also promotes oxidative burst in healthy human neutrophils (Figure 2). Indeed, neutrophils were able to secrete ROS in the presence of *T. rubrum* hyphae or conidia. Interestingly, when co-cultures were treated with diphenyleneiodonium chloride (DPI), ROS production was significantly reduced, indicating that most of these reactive species are derived from the NADPH oxidase complex (Figure 2A). Again, fungal viability was not required for oxidative burst since heat-killed (HK) morphotypes still retained their stimulatory capacity (Figure 2B).

### 2.3. NET Induction by T. rubrum Is Dependent on ROS Production

**Figure 3:**
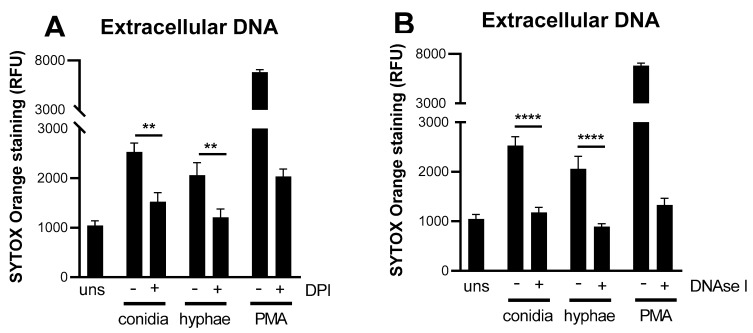
NET formation by human neutrophils against *T. rubrum* is dependent on ROS production. Neutrophils were pre-treated with DPI or DNase I before stimulation with *T. rubrum* morphotypes and incubated for 3 h. Extracellular DNA was quantified using SYTOX Orange by measuring its fluorescence intensity (expressed as relative fluorescence units, RFU). (**A**) Effect of DPI treatment on NET induction. (**B**) Effect of DNase I. Data shown as the mean ± SEM of three independent experiments. One-way ANOVA and Bonferroni’s post-test: ** *p* < 0.01; **** *p* < 0.0001.

Previous reports showed that NETs can be a downstream result of ROS production and inhibition of NADPH oxidase with DPI effectively blocks their induction [17]. Thus, we investigated whether *T. rubrum*-induced NETs were ROS dependent (Figure 3). As expected, neutrophils treated with DPI and infected with *T. rubrum* showed a significant reduction in NETs independent of the morphotype (Figure 3A). A similar reduction was also observed when the cells were treated with DNase I (Figure 3B), suggesting that the detected extracellular DNA was indeed part of a NET complex.

### 2.4. NET-Mediated Killing of T. rubrum

**Figure 4:**
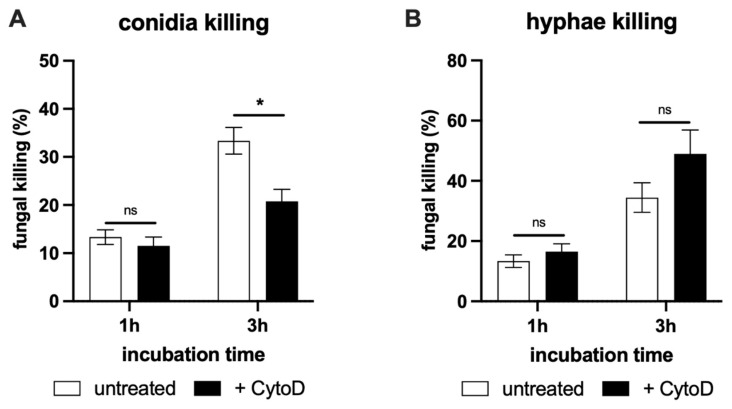
NETs can entrap and kill *T. rubrum***.** (**A**) Conidia or (**B**) hyphae were incubated with neutrophils for the indicated periods with or without cytochalasin D. Fungal viability was tested by the MTT-based colorimetric approach, and the reduction in viable fungi was expressed as the percentage of fungal killing. Data shown as the mean ± SEM. One-way ANOVA and Bonferroni’s post-test: ns, not significant; * *p* < 0.05.

Having established the ability of neutrophils to release NETs in response to *T. rubrum* stimulation, we inquired what their contribution to fungal killing is. Since neutrophils can perform phagocytosis and intracellular killing of fungal pathogens, we treated the cells with the actin inhibitor cytochalasin D to suppress internalization and, therefore, to be able to evaluate the extracellular killing separately (Figure 4).

We observed that neutrophils were more efficient in hyphae elimination, displaying almost 50% higher killing potential than observed for conidia in the first hour of interaction. In addition, hyphae assays were not affected by cytochalasin D, indicating NETs might be the main pathway for this morphotype killing (Figure 4B). Strikingly, conidia inactivation was significantly reduced by phagocytosis blockage, suggesting that intracellular killing is an important component in conidia elimination and may act in synergy with NET formation (Figure 4A). Thus, even though *T. rubrum* conidia can trigger NETs, neutrophils still rely on active internalization for effective spore restriction.

Our results indicated that *T. rubrum* is an efficient NET inducer despite its morphotypes. However, while NETs play the most relevant role in hyphae restriction, conidia elimination also involves phagocytosis of fungal particles.

## 3. Discussion

The increased incidence of invasive fungal infections, especially due to the development of efficient immunosuppressive therapies, highlights the importance of the understanding of the immune response against fungi. The identification of deep dermatophytosis cases renewed the interest in the study of immunity to dermatophytes. Here, we showed that neutrophils can generate NETs in response to *T. rubrum*, and they might play a key role in hyphae clearance. Some studies have already shown that several medically important fungi, including *C. albicans*, *Aspergillus* spp., and the dermatophyte *Arthroderma benhamiae*, have the ability to induce NETs [11,17,18,19,20], and here, we add *T. rubrum* to this growing list.

It is still not fully understood how neutrophils determine which effector response will they choose once challenged. Branzk et al. [21] suggested that neutrophils sense microbial size and selectively release NETs in response to large pathogens, such as *C. albicans* hyphae and extracellular *Mycobacterium bovis* aggregates, but not to small yeast and single bacteria. Nonetheless, this rule does not apply to *T. rubrum* since both conidia and hyphae were NET inducers, suggesting that other local clues are also involved. Interestingly, even though NETs are mobilized, they may not contribute to pathogen elimination. Thus, although *T. rubrum* conidia promote NET response, they still require engulfment-associated mechanism. Similarly, it was shown that *Aspergillus* sp. is a NET inducer, but its elimination does not rely on this response either [17].

It is accepted that NET initiation may be broadly classified into ROS-dependent and -independent pathways [18,22,23,24]. Most of the ROS-dependent stimuli are NADPH oxidase activators, including here *T. rubrum*. Yet, other ROS inducers such as *Staphylococcus aureus*, *C. albicans* and ionomycin were shown to be ROS-independent NET triggers [20,23,25]; thus, the ROS generating potential cannot be considered the sole variable. In addition, it was also curious to observe that heat-inactivated *T. rubrum* is still able to promote NET release, similar to observations made for *P. verrucosa* and the apicomplexan parasite *Besnoitia besnoiti* [20,26], suggesting that NET inducers are heat-stable molecules and are not related to pathogen viability. Instead of a unique molecular pattern, it is likely that the NET activators might share common signaling cascades, similar to what is observed for the NLRP3 inflammasome, which responds to structurally diverse molecules [27].

Overall, our data provided novel evidence that *T. rubrum* can induce NET formation through ROS production and that this response might be the main mechanism for hyphae killing. Further studies are required to better understand how redox mechanisms triggered by *T. rubrum* lead to NET formation and what is the contribution of this response in vivo.

## 4. Material and Methods

### 4.1. Fungi

A clinical isolate of *T. rubrum* (IMT-20) was used in this study. Fungi were grown for ≥15 d on Sabouraud agar tubes (BD Biosciences, Sparks, USA) at 30 °C until sporulation was sufficient. Conidia and hyphae were collected by washing with 0.9% NaCl solution and filtered through 40 μm cell strainers (BD Biosciences, Sparks, USA). Conidial particles were obtained from the flow-through solution, while hyphae were retained in the cell strainer. After 3 washes with saline solution, conidia were counted and diluted to the desired concentrations. For hyphae, the fungal structures retained in the cell strainers were washed extensively with 0.9% NaCl, and recovered hyphae fragments were counted and diluted to the desired concentrations. Conidial particles could not be totally removed from hyphae suspensions, and their proportion in hyphae suspensions was 3 conidia for 1 hypha. Heat-killed *T. rubrum* were prepared by incubating the inoculum in a dry-bath at 70 °C for 1 h. Inactivation was confirmed platting 0.2 mL of fungal suspensions on Sabouraud dextrose agar plates.

### 4.2. Human Neutrophil Collection

Blood was obtained from with participants’ consent through the protocol approved by the ethical committee of Clinics Hospital, University of São Paulo (Approval #065235/2018). Blood samples were collected from ten healthy adult volunteers (6 males, 4 females, mean age of 35 y) who were free of infectious or inflammatory diseases at the moment of sample acquisition. Blood was collected in heparin-containing Vacutainer tubes (BD Biosciences, Sparks, NV, USA). Neutrophils were obtained by dextran sedimentation, followed by Ficoll-Hypaque (GE Healthcare, Chicago, IL, USA centrifugation, as previously described by Quach and Ferrant [28]. The viability and purity of fresh isolated neutrophils were consistently greater than 95%, as determined by Trypan blue exclusion.

### 4.3. Immunofluorescence of NETs

As previously described by Vong et al. [29], human neutrophils were adhered onto glass coverslips in 24-well non-treated culture plates by incubation for 30 min at 37 °C. They were then inoculated with *T. rubrum* conidia or hyphae and incubated for an additional 3 h at 37 °C. For the positive control, 100 nM phorbol myristate acetate (PMA) was added to stimulate neutrophils. Monolayers were fixed with 4% paraformaldehyde (Labsynth, Diadema, Brazil) in PBS (Sigma-Aldrich, Burlington, VT, USA), permeabilized with 0.1% Triton X-100 (Sigma-Aldrich, Burlington, VT, USA), and blocked with 3% albumin bovine serum (BSA), (Sigma-Aldrich, Burlington, VT, USA). Samples were incubated with primary antibodies: mouse monoclonal anti-myeloperoxidase (AB25989, Abcam, Cambridge, UK) and rabbit polyclonal anti-histone H3 (AB5103, Abcam, Cambridge, UK), followed by exposure to a secondary antibody conjugated to Alexa Fluor 488 (ab150113, Abcam, Cambridge, UK) or Alexa Fluor 568 (ab175471, Abcam, Cambridge, UK), and nuclear staining was made with Hoechst dye. Cells were analyzed by fluorescence microscopy in a Nikon eclipse 80i.

### 4.4. NETs (DNA/MPO) Quantification

Myeloperoxidase-deoxyribonucleic acid (MPO-DNA) complexes were measured in supernatants as previously described by Colón et al. (2019). Briefly, anti-myeloperoxidase antibody (AB25989) (5 μg/mL; Abcam, Cambridge, UK) was bound to a 96-well clear-bottom plate, and it captured MPO in the supernatants. The amount of DNA complexed to MPO was quantified using the Quant-iT™ PicoGreen^®^ kit (Invitrogen, Waltham, MA, USA) according to the manufacturer’s instructions. The fluorescence intensity (excitation at 488 nm and emission at 525 nm) was quantified in a FlexStation 3 Microplate Reader (Molecular Devices, San Jose, CA, USA).

In addition, NET formation was also indirectly quantified by SYTOX Orange (Life Technologies, Carlsbad, CA, USA) staining of extracellular DNA, and the results are expressed in relative fluorescence units (RFU).

### 4.5. Luminol-Amplified Chemiluminescence Assay

Neutrophils (2 × 10^5^/mL) were suspended in RPMI without phenol in the presence of luminol (10 nM). Cells were then stimulated with *T. rubrum* conidia, hyphae (MOI 1:5), or PMA (100 nM) for 2 h at 37 °C. For NADPH oxidase blockade, wells were treated with 16 µM DPI. The total ROS production was determined by chemiluminescence. The results are expressed as relative light units (RLU) during the observation period.

### 4.6. Assessment of NET-Mediated T. rubrum Killing

To determine the efficiency of NETs in killing *T. rubrum*, a tetrazolium salt MTT assay was employed. Fresh human neutrophils were incubated with *T. rubrum* conidia or hyphae in 96-well culture plates for 1 h and 3 h at MOI 1:5. For phagocytosis blockade, wells were treated with 10 µg/mL cytochalasin D. At each timepoint, the numbers of viable fungi were determined by lysing the neutrophils with water, and the suspensions were incubated with yellow tetrazolium salt 3-(4,5-dimethylthiazol-2-yl)-2,5-diphenyltetrazolium bromide (MTT) for 3 h. The hydrogenases of viable fungi cleaved MTT to its purple derivative, MTT-formazan, which could be quantified by spectrophotometry. The reduction of MTT-formazan formation is expressed as % of killing.

### 4.7. Statistical Analysis

Data are expressed as the mean ± SEM and were analyzed in the software GraphPad Prism (Version 9.3 for Windows, GraphPad Software, San Diego, CA, USA, www.graphpad.com (accessed on 21 January 2022). A *p*-value < 0.05 was considered statistically significant. The number of replicates and the appropriate test are disclosed in each figure legend.

## Data Availability

The datasets generated for this study are available upon request to the corresponding author.

## References

[1] Achterman R.R., White T.C. (2012). Dermatophyte virulence factors: Identifying and analyzing genes that may contribute to chronic or acute skin infections. Int. J. Microbiol..

[2] Nir-Paz R., Elinav H., Pierard G.E., Walker D., Maly A., Shapiro M., Barton R.C., Polacheck I. (2003). Deep infection by Trichophyton rubrum in an immunocompromised patient. J. Clin. Microbiol..

[3] Rouzaud C., Chosidow O., Brocard A., Fraitag S., Scemla A., Anglicheau D., Bouaziz J.D., Dupin N., Bougnoux M.E., Hay R. (2018). Severe dermatophytosis in solid organ transplant recipients: A French retrospective series and literature review. Transpl. Infect. Dis..

[4] Romero F.A., Deziel P.J., Razonable R.R. (2011). Majocchi’s granuloma in solid organ transplant recipients. Transpl. Infect. Dis..

[5] Papayannopoulos V. (2018). Neutrophil extracellular traps in immunity and disease. Nat. Rev. Immunol..

[6] Drummond R.A., Gaffen S.L., Hise A.G., Brown G.D. (2014). Innate Defense against Fungal Pathogens. Cold Spring Harb. Perspect. Med..

[7] Hogan D., Wheeler R.T. (2014). The complex roles of NADPH oxidases in fungal infection. Cell Microbiol..

[8] Brinkmann V., Reichard U., Goosmann C., Fauler B., Uhlemann Y., Weiss D.S., Weinrauch Y., Zychlinsky A. (2004). Neutrophil extracellular traps kill bacteria. Science.

[9] Rocha J.D., Nascimento M.T., Decote-Ricardo D., Côrte-Real S., Morrot A., Heise N., Nunes M.P., Previato J.O., Mendonça-Previato L., DosReis G.A. (2015). Capsular polysaccharides from Cryptococcus neoformans modulate production of neutrophil extracellular traps (NETs) by human neutrophils. Sci. Rep..

[10] Guimaraes-Costa A.B., Nascimento M.T., Froment G.S., Soares R.P., Morgado F.N., Conceicao-Silva F., Saraiva E.M. (2009). Leishmania amazonensis promastigotes induce and are killed by neutrophil extracellular traps. Proc. Natl. Acad. Sci. USA.

[11] Urban C.F., Nett J.E. (2019). Neutrophil extracellular traps in fungal infection. Semin. Cell Dev. Biol..

[12] Della Coletta A.M., Bachiega T.F., de Quaglia e Silva J.C., Victoriano de Campos Soares A.M., De Faveri J., Marques S.A., Alencar Marques M.E., Ximenes V.F., Dias-Melicio L.A. (2015). Neutrophil Extracellular Traps Identification in Tegumentary Lesions of Patients with Paracoccidioidomycosis and Different Patterns of NETs Generation In Vitro. PLoS Negl. Trop. Dis..

[13] Alflen A., Aranda Lopez P., Hartmann A.K., Maxeiner J., Bosmann M., Sharma A., Platten J., Ries F., Beckert H., Ruf W. (2020). Neutrophil extracellular traps impair fungal clearance in a mouse model of invasive pulmonary aspergillosis. Immunobiology.

[14] Byrd A.S., O’Brien X.M., Johnson C.M., Lavigne L.M., Reichner J.S. (2013). An extracellular matrix-based mechanism of rapid neutrophil extracellular trap formation in response to Candida albicans. J. Immunol..

[15] Mejía S.P., Cano L.E., López J.A., Hernandez O., González Á. (2015). Human neutrophils produce extracellular traps against Paracoccidioides brasiliensis. Microbiology.

[16] de Sousa M.d.G.T., Santana G.B., Criado P.R., Benard G. (2015). Chronic widespread dermatophytosis due to Trichophyton rubrum: A syndrome associated with a Trichophyton-specific functional defect of phagocytes. Front. Microbiol..

[17] Gazendam R.P., van Hamme J.L., Tool A.T., Hoogenboezem M., van den Berg J.M., Prins J.M., Vitkov L., van de Veerdonk F.L., van den Berg T.K., Roos D. (2016). Human Neutrophils Use Different Mechanisms To Kill Aspergillus fumigatus Conidia and Hyphae: Evidence from Phagocyte Defects. J. Immunol..

[18] Bianchi M., Hakkim A., Brinkmann V., Siler U., Seger R.A., Zychlinsky A., Reichenbach J. (2009). Restoration of NET formation by gene therapy in CGD controls aspergillosis. Blood.

[19] Heddergott C., Bruns S., Nietzsche S., Leonhardt I., Kurzai O., Kniemeyer O., Brakhage A.A. (2012). The Arthroderma benhamiae hydrophobin HypA mediates hydrophobicity and influences recognition by human immune effector cells. Eukaryot. Cell.

[20] Liu Q., Yi W., Jiang S., Song J., Liang P. (2021). Neutrophil Extracellular Traps Serve as Key Effector Molecules in the Protection Against Phialophora verrucosa. Mycopathologia.

[21] Branzk N., Lubojemska A., Hardison S.E., Wang Q., Gutierrez M.G., Brown G.D., Papayannopoulos V. (2014). Neutrophils sense microbe size and selectively release neutrophil extracellular traps in response to large pathogens. Nat. Immunol..

[22] Fuchs T.A., Abed U., Goosmann C., Hurwitz R., Schulze I., Wahn V., Weinrauch Y., Brinkmann V., Zychlinsky A. (2007). Novel cell death program leads to neutrophil extracellular traps. J. Cell Biol..

[23] Parker H., Dragunow M., Hampton M.B., Kettle A.J., Winterbourn C.C. (2012). Requirements for NADPH oxidase and myeloperoxidase in neutrophil extracellular trap formation differ depending on the stimulus. J. Leukoc. Biol..

[24] Wu S.Y., Weng C.L., Jheng M.J., Kan H.W., Hsieh S.T., Liu F.T., Wu-Hsieh B.A. (2019). Candida albicans triggers NADPH oxidase-independent neutrophil extracellular traps through dectin-2. PLoS Pathog..

[25] Pilsczek F.H., Salina D., Poon K.K., Fahey C., Yipp B.G., Sibley C.D., Robbins S.M., Green F.H., Surette M.G., Sugai M. (2010). A novel mechanism of rapid nuclear neutrophil extracellular trap formation in response to Staphylococcus aureus. J. Immunol..

[26] Muñoz Caro T., Hermosilla C., Silva L.M., Cortes H., Taubert A. (2014). Neutrophil extracellular traps as innate immune reaction against the emerging apicomplexan parasite Besnoitia besnoiti. PLoS ONE.

[27] Swanson K.V., Deng M., Ting J.P. (2019). The NLRP3 inflammasome: Molecular activation and regulation to therapeutics. Nat. Rev. Immunol..

[28] Quach A., Ferrante A. (2017). The Application of Dextran Sedimentation as an Initial Step in Neutrophil Purification Promotes Their Stimulation, due to the Presence of Monocytes. J. Immunol. Res..

[29] Vong L., Sherman P.M., Glogauer M. (2013). Quantification and visualization of neutrophil extracellular traps (NETs) from murine bone marrow-derived neutrophils. Methods Mol. Biol..

